# Strengthening Health Care Professionals’ Collaborative Responses to Women Experiencing Intimate Partner Violence in Pregnancy: Protocol for an Exploratory Mixed Methods Study

**DOI:** 10.2196/86289

**Published:** 2026-03-24

**Authors:** Carly Jones, Belinda Lovell, Tracy Humphrey, Angela Brown

**Affiliations:** 1 College of Health, Adelaide University, Corner of North Terrace and Frome Road, Adelaide, SA, 5001, Australia, 61 83027733; 2 College of Nursing and Health Sciences, Flinders University, Adelaide, SA, Australia

**Keywords:** intimate partner violence, domestic violence, collaboration, health professional education, interdisciplinary, simulation-based learning

## Abstract

**Background:**

Effective collaborative practice among health care professionals is crucial for addressing intimate partner violence (IPV) during pregnancy. Therefore, the development and evaluation of an evidence-based intervention for health care professionals is required to work toward meeting the key priorities of the National Plan to End Violence Against Women and Children 2022‐2032. The consistency, modality, and effectiveness of IPV-focused education vary, and some midwives lack the confidence to respond effectively to disclosures, often due to limited knowledge, education, and skills. This issue is further amplified in interdisciplinary settings, where a lack of cohesiveness and collaboration can negatively impact the experience for pregnant women seeking or needing support.

**Objective:**

The objective of this study is to design, implement, and evaluate an evidence-informed, simulation-based, interprofessional education intervention to improve health care professionals’ collaborative response to IPV disclosures during the perinatal period in the South Australian maternity health care sector.

**Methods:**

This study adopts an exploratory mixed methods research design with 3 key phases. Phase 1 explores the experiences of women with lived experience of IPV in pregnancy (a minimum of 9 participants), and health care professionals providing perinatal care (4‐8 participants in approximately 4‐8 focus group offerings). The data will inform the development of the educational program. Phase 3 focuses on the evaluation of the intervention to determine the perceived impact. There will be several offerings of the workshops to capture a minimum of 42 health professional participants.

**Results:**

Qualitative data will be collected first from interviews with women with lived experience of IPV in the perinatal period and focus groups with health professionals who have provided care across that context. Phase 3 comprises quantitative survey data through a single-group quasi-experimental pretest-posttest design to determine the perceived impact of the workshop.

**Conclusions:**

This study aims to provide insights into the effectiveness of educational interventions designed to enhance collaboration within interdisciplinary teams in the context of IPV.

## Introduction

### Background

Intimate partner violence (IPV) is a long-standing global phenomenon that transcends cultural, social, and economic boundaries, affecting millions of women and their families [1]. IPV is defined as behaviors within an intimate relationship (current or previous) whereby there is physical, sexual, or psychological harm [2]. Coercive control, however, is the main context in which IPV presents [3]. The difference between individual and societal perspectives on how IPV is defined ultimately shapes how the prevalence, patterns, and health consequences of IPV are understood and measured [4]. In fact, the true extent of IPV may not be accurately reflected in current statistics due to the structural and systemic factors that contribute to underreporting [5,6].

In Australia, current statistics report that IPV has accounted for 57 domestic homicide deaths of women between 2023 and 2024 and has affected approximately 1 in 4 women and girls from the age of 16 [7-10]. Pregnancy does not serve as a protective factor for IPV as it has been shown to begin or persist throughout this time, often acknowledged as a risk factor for escalating to death across this period [11,12]. Exposure to IPV while pregnant is associated with poorer maternal and neonatal health outcomes, such as increased likelihood of suicide, emotional distress, miscarriage, stillbirth, preterm labor, and low birth weight [13]. The profound health implications of IPV during pregnancy extend beyond immediate physical harm, contributing to long-term maternal and neonatal morbidity, increased health care costs, and greater strain on health care systems; this highlights the urgent need for targeted intervention and support [13].

The perception of what constitutes IPV is shaped by individual, relational, and structural factors [1]. The complexity of IPV is further exacerbated by health disciplines having varying perceptions of IPV due to the differences in their key responsibilities, organizational structures, and institutional practices [14]. Limited knowledge and understanding of the cross-disciplinary roles and responsibilities is a well-known factor that hinders collaborative responses to support women [15,16]. While institutional and organizational barriers often influence collaboration, by focusing on improving the understanding of cross-discipline roles and individual responsibilities, it can have a considerable impact on collaboration [14,17].

Several programs and interventions have been implemented and evaluated that focused on addressing the challenges and deficits of health professionals in providing care to women experiencing IPV in pregnancy [18-20]. While these programs have had a positive effect on increasing health professionals’ knowledge, the effectiveness could be further enhanced through an interprofessional approach [18-20]. While there are existing programs that have an interprofessional approach, they did not target health professionals actively working with women experiencing IPV; instead, they focused on undergraduate students [19,21].

Workforce training and education were emphasized as a key priority in the National Plan to End Violence Against Women and Children 2022‐2032 report [1]. While a deficit in available IPV-focused education for health professionals has been acknowledged, the problem is further exacerbated by the continual siloed approach of education [21]. When education is offered in a discipline-specific approach, health professionals remain underprepared to work in a multidisciplinary team to address the complex social issues associated with IPV presentations in pregnancy [21]. To achieve meaningful improvements in maternal and neonatal outcomes, it is essential to implement and evaluate IPV educational programs that focus on collaboration among health care professionals [13].

This study aims to develop a workshop that includes the voices of women with lived experience of IPV across the perinatal period and the health professionals who provide the care. The purpose is to integrate their needs, perspectives, and experiences to capture the current context of perinatal care in South Australia and inform the development of a bespoke workshop. This paper describes the study protocol for the development and evaluation of an interprofessional, IPV-focused simulation workshop for health professionals to develop the necessary skills to collaboratively care for women experiencing IPV and accessing health care in the perinatal period.

### Aim

The aim of this study is to design, implement, and evaluate an evidence-informed, simulation-based, interprofessional education intervention to improve health care professionals’ collaborative response to IPV disclosures and ongoing management of care during the perinatal period in the South Australian maternity health care sector.

### Objectives

The objectives of the study are as follows:

1. To design and develop a simulation workshop for health professionals that captures the experiences of women with lived experience of IPV in pregnancy in addition to addressing the needs of health professionals providing that care.
2. To evaluate the perceived impact of the simulation workshop to determine the changes in the health professional’s collaboration and behavioral response to IPV disclosures and ongoing management of care throughout the perinatal context.
3. To evaluate changes in participants’ objective knowledge related to recognizing and responding to IPV disclosures during maternity care following a simulation-based interprofessional education intervention, using standardized pre- and postintervention knowledge measures.
4. To evaluate changes in participants’ interprofessional responses to simulated IPV disclosure scenarios, using standardized assessment that is scored with predefined rubrics before and after the intervention.

### Research Questions

The research questions of the study are as follows:

1. What are the key components of an evidence-informed simulation-based interprofessional education intervention?
2. What interprofessional IPV education and training is accessible for health care providers working in the maternity sector within their workplaces and how do they perceive its relevance and effectiveness?
3. What are the experiences and perceptions of engaging with maternity care among women with lived experience of IPV in South Australia?
4. How does simulation-based interprofessional education intervention influence health care professionals’ collaboration, knowledge, and behavioral response to IPV disclosures in the perinatal context?

## Methods

### Study Design

This proposed study will use an exploratory mixed methods research design to collect and analyze qualitative data. Phase 1 explores the perspectives and experiences of victim-survivors and health care providers in 2 separate data collection modalities. This data will guide the development of the educational workshop, created during phase 2. Phase 3 will therefore involve the evaluation of the simulation education workshop, using a pretest-posttest design, through the integration of the 16-question Interprofessional Education Collaborative (IPEC) competency self-assessment which has been tested for validity, reliability, and usability [22].

### Philosophical Framework

**Figure 1.**
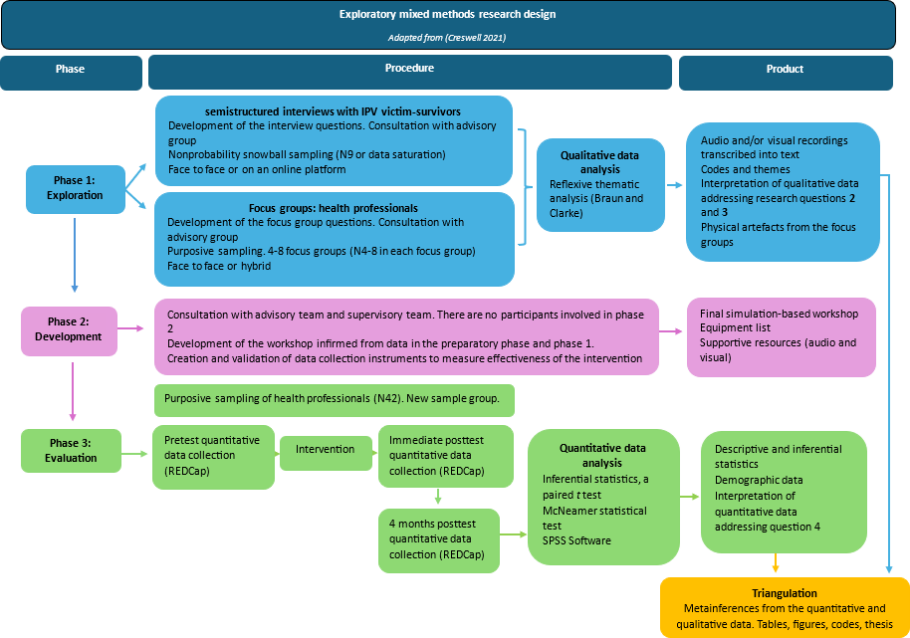
Phases of the proposed exploratory mixed methods research design. IPV: intimate partner violence; REDCap: Research Electronic Data Capture.

The pragmatic philosophy allows for methodological flexibility in approach and provides a comprehensive insight into the problem [23] (Figure 1). Pragmatic research often integrates a mixed methods approach to first identify a problem and collect multiple forms of data from varying cohorts of participants and then apply the findings in a practical method. This approach encourages researchers to remain open to a variety of assumptions and worldviews, allowing for a holistic and deeper understanding of the phenomenon [23]. The main aim is to develop an experience-driven, action-oriented program [24,25].

### Exploratory Mixed Methods

The project integrates an exploratory mixed methods research design, which involves the collection and integration of qualitative data, followed by quantitative data [23,26,27]. The aim of doing so is to develop a deeper understanding of the phenomena by integrating multiple perspectives through differing data subsets [23,26]. The phase 1 qualitative findings inform the phase 3 quantitative instruments. Dawadi et al [27] note that an exploratory process is important when the research aims to focus on transferability and generalizability. The development of the quantitative instruments in phase 3 of this research is developed following the phase 1 findings, thus consistent with exploratory mixed methods research. The application of mixed methods research in health is an appropriate methodology due to the improved applicability and greater insight generated (depth and breadth) [23,26,27].

### Setting

Women with lived experience of IPV will be sought from regional and metropolitan South Australia. The health professionals will be accessed through the same locations; however, they must be practicing in the public or private maternity sector and providing perinatal health care to women and their families. The physical environment, particularly the lived experience interviews, will need to be safe for both the first author and participant and will be taken into consideration. The evaluation of the project will be set at Adelaide University campus in the simulation spaces.

### Sampling

The interviews will integrate a nonprobability snowball sampling technique to capture women with lived experience; a useful sampling technique to engage with populations that present considerable recruitment challenges [28]. This process will be initiated through initial contact with experts and domestic violence organizations.

The focus group participants (phase 1) and evaluation of the workshop (phase 3) will use purposive sampling techniques to ensure a broad representation of disciplines [29].

### Participants

Phase 1 has 2 different focuses of participants: women with lived experience of IPV and a minimum of 5 different health profession disciplines. Phase 3 will integrate a new cohort of health professionals.

#### Phase 1: Lived Experience Participants

Inclusion criteria require people with lived experience of IPV, including any classification such as physical, sexual, or psychological [30,31]. They must have had a pregnancy and accessed maternity care in the past 5 years and be 18 years or older. Participants who are currently pregnant at the time of the interview will be excluded.

#### Phase 1: Health Professional Participants

For eligibility, participants must be currently practicing in a health profession that is likely to have managed care for pregnant women who have experienced IPV. The health professionals that are most likely to meet this criterion are midwives, social workers, medical staff, perinatal mental health nurse midwives, and Aboriginal Maternal and Infant Care workers (refer to Table 1 for inclusion and exclusion criteria).

**Table 1.** Inclusion and exclusion criteria for health professionals.

Discipline	Inclusion	Exclusion
Midwives	Registered with the Ahpra^a^ as a midwife and currently working in the clinical environment and has provided care to women who have experienced and disclosed IPV^b^ in pregnancy in the past 5 years.	Does not hold registration with Ahpra as a midwife or has conditions as part of their registration and/or has not provided care to women who have experienced and disclosed IPV in pregnancy in the past 5 years.
Social workers	Registered with Ahpra as a social worker and currently practicing clinically and has experience with working with women with lived experience of intimate partner violence in pregnancy in the past 5 years.	No longer practicing clinically or not registered with Ahpra as a social worker and/or does not have experience working with women with lived experience of intimate partner violence in pregnancy in the past 5 years.
Medical staff	Registered with Ahpra as a medical practitioner and undertaken an obstetric rotation in the past 5 years. Additionally, has provided care to women who have experienced and disclosed IPV in pregnancy.	Is not registered with Ahpra as a medical practitioner or has never practiced in obstetrics or has not practiced in the past 5 years. Additionally, if they have not provided care to women who have experienced and disclosed IPV in pregnancy.
Perinatal mental health nurse midwives	Registered with Ahpra as a nurse and/or midwife and currently practicing clinically. Has provided care to women who have experienced and disclosed IPV in pregnancy in the past 5 years. A postgraduate qualification in perinatal mental health has been obtained.	Does not hold registration with Ahpra as a nurse and/or midwife or has conditions as part of their registration and/or has not provided care to women who have experienced and disclosed IPV in the past 5 years. A graduate qualification in perinatal mental health has not been obtained.
AMIC^c^ Workers	Have experience working with women with lived experience of intimate partner violence in pregnancy in the past 5 years.	Does not have experience working with women with lived experience of intimate partner violence in pregnancy in the past 5 years.

a Ahpra: Australian Health Practitioner Regulation Agency.

b IPV: intimate partner violence.

c AMIC: Aboriginal Maternal and Infant Care.

#### Phase 3: Participants

The disciplines will remain the same as phase 1 health professionals; however, participants do not need to have had experience working with women who experienced IPV.

The disciplines included in phase 3 will remain iterative, as the earlier phases may capture additional disciplines who care for women with lived experience of IPV and may benefit from the proposed research.

### Recruitment

#### Phase 1

Self-referral rates among IPV victim-survivors for research participation are often low [32]. Recruitment of lived experience participants will therefore occur through domestic violence organizations and networks with health professionals who are working with women impacted by IPV [33,34]. The chief investigator will email experts expressing the purpose of the research [2]. Those experts who then agree to assist with recruitment will be asked to identify and refer other potential participants who meet the eligibility criteria, thereby extending recruitment of victim-survivors through their established networks [35].

For inclusion in the focus groups, health professionals will be recruited through the Local Health Networks in South Australia and connections with potential end users of the program [29]. Social media platforms such as Facebook (Meta Platforms, Inc) and LinkedIn (Microsoft Corp) will be used for recruitment, given their cost-effectiveness and ability to reach a broad audience [36]. Furthermore, flyers will be disseminated to management personnel at the Local Health Networks to support recruitment and dissemination.

#### Phase 3

Phase 3 recruitment of health professionals will be conducted using the same method as phase 1.

### Ethical Considerations

#### Approval

For this research, several ethics applications are required. Phase 1 lived experience interviews received ethics approval from the University of South Australia Human Research Ethics Committee (207340) in November 2025. The research to be undertaken for the focus groups and workshop evaluation (phase 1 and phase 3) will require ethics approval from the Department for Health and Wellbeing Human Research Ethics Committee, and site-specific approval for the Local Health Networks in South Australia [37,38]. Following approval, ratification through the Adelaide University Human Research Ethics Committee (formerly known as the University of South Australia Human Research Ethics Committee) will be sought. Phase 2 is the development of the project and has no engagement with participants, thus not requiring ethics approval.

#### Informed Consent

All participants across each phase will be provided with a detailed explanation of the participation requirements and expected commitment during the recruitment period [39]. This participant information sheet (PIS) will provide details of the study, requirements of participation, data management, reimbursement, and the consent process. All participants can opt to access the PIS in either a digital or hard copy version. Consent will be specific and limited to the phase of engagement in the proposed project. Participants will also be informed through the PIS that in the event of disclosures indicating a current risk of harm to the participant or child, the researchers have a legal obligation to take appropriate action. Where there is immediate danger to a child and/or participant, the interview will be ceased, and emergency services (000 or 131 444) will be contacted. Where there is a potential risk, however, no immediate danger to a child, the researcher will notify the relevant child protection authority. The consent form requires participants to acknowledge that they have read the PIS as part of the consent process.

Prior to signing the consent form, participants will be asked to complete the self-reflection checklist developed by Lamb et al (refer to Multimedia Appendix 1) [40]. This checklist is designed to ensure that participants engage in self-reflection, are well-informed, and feel adequately prepared to participate in the study [40]. If participants determine they are not prepared to undertake the research after completing the reflective questions, they will be excluded from participation and offered referrals and access to support guided by the study distress protocol [40]. Participants will have the opportunity to ask questions that arise from the lived experience self-reflection checklist to gain clarity and support or can be referred to the support services outlined in the distress protocol (refer to Multimedia Appendix 2) [40].

#### Gratuity

The inclusion of lived experience participants without payment is often considered exploitative [41,42]; yet, there remains inconsistency in the policy regarding ethical payment in research for lived experience [41]. Interview participants will be offered reimbursement for the costs incurred during participation (a $50 VISA giftcard), in alignment with the recommendations outlined in the National Statement on Ethical Conduct in Human Research (National Health and Medical Research Council [NHMRC]) 2025 policy [43]. Participants in the focus groups are voluntary.

#### Participant Safety and Withdrawal

All participants will have the right to withdraw at any time during the study, up until the analysis of the data. The health professionals will be informed that after completion of the focus groups, their data will be unable to be removed due to the nature of focus group research.

Women with lived experience of IPV will not be directly recruited through the research team. At no stage will the chief investigator directly reach out to potential participants. Instead, to protect women’s safety, snowball sampling techniques through agencies and experts in this field will occur to facilitate recruitment. This indirect way of recruiting aims to prevent coercion. Participants may contact the primary investigator via their preferred method to express interest in the study. By initiating contact with the primary investigator through a participant-initiated method, they are assumed to consider this mode of communication safe. Upon providing the PIS, the primary investigator will verify with each participant that their preferred contact method is suitable and secure, using direct questioning to ensure safe communication.

The process of member checking for women with lived experience of IPV will provide the opportunity to review the full verbatim transcript and redact a statement if they wish. This process will occur via the participants' personal email or another method of communication of their choice. In the email, a summary of the main themes will be included if they choose not to read the entire transcript. Following the interview, they will also receive a follow-up phone call to check in on their well-being. The health professional participants from phase 1 will receive a phone call the following day to check on their well-being. They will also receive an email with the opportunity to clarify the themes and topics from a summary of the focus groups and interview findings. Prior to sending this information, the primary investigator will confirm with the participants that they consent to receive this information through the previously agreed communication method.

#### Data Management

All data obtained throughout the study will be stored in accordance with the NHMRC management of data guide which was developed in response to the Australian code of conduct of research [43]. All electronic recordings and data will be securely stored on a password-protected laptop (provided by the Adelaide University), OneDrive system, and USB. This system does not allow for external access and sharing, thus it meets the Australian data sovereignty standards [44]. Any physical documents obtained will be securely stored in a locked cabinet within a locked office at the University. Data will be stored in this manner for 7 years, adhering to the minimum 5 years from the first date of publication [43]. After this date, data will be securely destroyed.

### Engagement With Participants With Lived Experience of IPV

#### Overview

Women who have experienced IPV have an increased risk of adverse outcomes when participating in research [43]. The NHMRC recognize people who have experienced social and economic disadvantage, such as trauma and violence, may have potential limitations with their decision-making ability [43]. To mitigate and minimize these risks, consultation with end users, such as social workers from Women’s Safety Services SA, will occur prior to the interviews with phase 1 participants to ensure the questions, activities, and discussion is a trauma-informed and healing approach [40]. To ensure victim-survivors are central to the design and empowered by the process, the research will align with best practice principles for coproduced research with victim-survivors and use a framework developed by Lamb et al [40] to support ethical coproduction of research with vulnerable populations of women. These best practice principles align with the NHMRC principles of justice, respect, and beneficence [43].

#### Distress Protocol

A distress protocol has been developed to ensure the research team is able to respond to participant distress. The flowchart was developed in alignment with recommendations from Whitney and Evered [45], Draucker et al [46], and Labott et al [47] and includes resources and support services (refer to Multimedia Appendix 2). The development of the distress protocol was in collaboration with the advisory group and supervisory team. At the completion of engagement, all participants of each phase will receive a pamphlet with detailed immediate and ongoing psychological support services that are available to access in person and/or online (refer to Multimedia Appendix 3).

#### Advisory Group

An advisory group, formed through connections with potential end users, will provide guidance throughout the research with an aim to reduce the risk of causing harm for those with lived experience. Six monthly meetings are planned to discuss the proposed research methodologies and intervention. The purpose is to ensure the program supports the needs of women with lived experience in addition to promoting a safe space that protects the well-being of those with lived experience and health professionals throughout the research.

#### Confidentiality

During data collection, analysis, and reporting, the participants will be labeled using predefined codes (Midwife 1, Social Worker 1, and Woman 1). This process is known as the dominant approach to addressing confidentiality and will be applied during the 3 phases of the process: data collection, data cleaning, and dissemination [48]. The women with lived experience will be informed that generalized, descriptive characteristics will be collected, and information will not be identifiable in the reporting process. This is similar to the health professionals whereby their generalized characteristics, for example, occupation and experience will be included, but not identifiable to maintain confidentiality and anonymity [48]. Identifying information will be stored separately from the transcripts. A deidentified dataset will be analyzed and disseminated, aiming to protect the confidentiality and anonymity of participants [48]. The PIS form provided to focus group participants will have a statement that while confidentiality will be maintained from the researcher’s perspective, participants are also obligated to maintain confidentiality across the focus group discussions given the public nature of the forum.

#### Vicarious Trauma

Due to the nature of the research, there is potential for the researchers involved to experience vicarious trauma. Vicarious trauma can occur from burnout, traumatic stress, and compassion fatigue [49]. The research team will have access to the Employee Assistance Program in preparation for potential discomfort or in response to trauma experienced. All research members will be asked to reflect on their involvement and are aware of the potential challenges of working in this field and agree to embrace differences in perspectives. The supervisory team will have the flexibility to opt into the phases of the research to ensure their comfort, and feedback will be sought iteratively throughout the project to ensure nonmaleficence.

### Procedure

#### Phase 1: Exploration

##### Overview

The objective of the interviews with women with lived experience of IPV is to understand their perspectives of engaging with maternity care services in South Australia (research question 3). The themes emerging from the interviews will offer valuable insight and inform the development of the workshop, ensuring it reflects and addresses the needs of women with lived experience in South Australia. From there, the importance is shifted to exploring the current interprofessional education and training on IPV received by maternity care providers in South Australia, to then examine their perceptions of its relevance and effectiveness (addressing research question 2). The focus groups aim to cultivate an understanding of each discipline’s roles, responsibilities, and values in relation to collaboration with other disciplines when navigating care for women with lived experience or IPV in pregnancy.

##### Lived Experience Interviews

Semistructured interviews will be used to explore the experiences and perceptions of maternity care among women with lived experience of IPV. The semistructured interviews allow for deeper exploration and the provision of participant autonomy to deviate and explore emerging concepts that may arise throughout the interview and further enhance the understanding of the issues [34,50]. The interview guide will be developed following the data extraction of the scoping review and reviewed by an advisory group and the supervisory team.

##### Data Saturation: Interviews

It is expected that data saturation will occur after a minimum of 9‐10 interviews [51]. Braun and Clarke [52], however, suggest researchers avoid limiting data collection to a specific number, and therefore in this study, the continuation of interviews will occur until data richness is achieved, and there is notable repetition in the presenting themes and codes.

##### Health Professional Focus Groups

Focus groups are incorporated due to the known benefits of exploring a group of health professionals’ opinions, perspectives, and views to ensure the richness of data [53]. Focus groups are particularly important when exploring new insights and understanding poorly explored topics as communication can be enhanced through a process defined as sharing and comparing [54]. The questions and activities will be structured and planned, commencing with an introduction, transition, and key questions followed by a final activity or question [55]. A range of group activities will be conducted, such as asking the group to arrange a series of statements in a particular order acknowledging that a consensus may not always be reached. The purpose is to observe the insights that arise [56]. The focus groups will be facilitated by 2 members of the research team to reduce bias [55].

##### Data Saturation: Focus Groups

A total of 4‐8 focus groups is recommended to achieve data saturation, with approximately 4‐8 participants in each group [51,54].

##### Data Analysis

Data will be analyzed by the primary investigator following the 6 steps to reflexive thematic analysis by Braun and Clarke [52] to determine the patterns and themes from the data. These iterative steps will ensure the data is interpreted and represented meaningfully [52]. The qualitative data will be collected in phase 1 and recorded and transcribed verbatim (by the principal researcher for the victim-survivor interviews and potentially an external third party for the focus groups) for the purpose of identifying, analyzing, and acknowledging patterns that come from the data [52,57]. Analysis will begin with familiarization of the data through transcription and rereading the transcripts to develop a deeper understanding [52,58]. Initial codes will be generated from identified patterns in the data [52]. Reflexive thematic analysis does not require a team of researchers to review codes; instead, the coding process is an “organic” process and does not adhere to specific frameworks [59]. One team member will independently code all data and share the findings with the research team. To improve trustworthiness and confirmability of the data, small portions of the transcripts will be allocated to differing authors, to confer, reflect, provide feedback, and therefore ensure a consensus is reached [60]. Trustworthiness in qualitative research can be strengthened further through reflexive processes [60]. Credibility in this study will be maintained through reflexive journaling to acknowledge personal biases to maintain an objective stance in the collection and data analysis processes [60].

After coding, the initial codes are reevaluated due to the reflexive, iterative nature of the 6 steps described by Braun and Clarke [52] and overarching themes of the data will be sought and determined. The continual reflection and review of the codes and themes will ensure the data represents the whole picture [52]. Once refined, the themes are then clearly defined and titled to articulate the meaning of the data [52]. Finally, the analysis is reported in a manuscript to present to the wider community [52].

The reflexive thematic analysis of focus groups with health care professionals and the interviews with victim-survivors will occur separately. While both qualitative data collection occurs in the same phase, each has a differing purpose and thus the research and analysis will be undertaken and reported separately.

### Phase 2: Development of the Advancing Learning in Gender-Based Violence Through Interprofessional Networks Workshop

The simulation-based workshop, referred to as “Advancing Learning in Gender-Based Violence through Interprofessional Networks” (ALIGN), will be developed following the phase 1 qualitative findings, as part of phase 2 of the broader project. The proposed structure of the day will incorporate 4 IPV scenarios, each with a differing focus. The structure will follow best practice standards, outlined by The International Nursing Association for Clinical Simulation and Learning Standards Committee, through the incorporation of structured learner activities, including a prebrief, simulation, and debrief format to ensure transferability and consistency of the program [61].

Simulation education through role play and facilitated discussions is known to improve the participants' knowledge in the clinical environment [58,62]. The proposed simulation-based education program will follow recommendations from the World Health Organization (WHO) through the development of learning objectives that are in alignment with their key recommendations for IPV-focused education for health professionals (clinical skills, resources, behaviors, and values) [58,63]. However, as the program aims to also influence health care professionals’ collaboration, the overall program objectives will be combined with the interprofessional competencies as defined by IPEC: values and ethics, roles and responsibilities, communication, teams, and teamwork [64]. This will support the effective delivery of the simulation workshops intended objectives, which are as follows:

1. Apply their own professional role and responsibilities in the clinical scenario, as well as describe the professional role and responsibility of other health professionals when responding to IPV.
2. To improve generalized knowledge of violence against women in the perinatal context and recognizing it as a significant public health concern.
3. Demonstrate ethical and women-centered care when responding, while acknowledging their own personal values and biases to actively collaborate with the interprofessional team.
4. Communicate in a trauma-informed, respectful manner with victim-survivors and interprofessional team members, sharing relevant information and providing feedback to support coordinated, safe, and supportive care.

### Phase 3: Evaluation

#### Overview

The evaluation of the ALIGN workshop will involve a single-group quasi-experimental pretest-posttest design (phase 3). The aim of phase 3 is to evaluate the perceived impact of the simulation-based interprofessional education intervention on health care professionals’ collaboration, knowledge, and behavioral responses to IPV disclosures during the perinatal period, addressing research question 4.

#### Data Collection Tools

A pre- and postquestionnaire will be used to assess the knowledge, attitude, and practice changes of participants. The preworkshop questionnaire will establish a baseline; the postworkshop questionnaire will therefore measure immediate effects on participant knowledge, attitude, practice, and behavior [65]. There will be another follow-up questionnaire at the 4-month postintervention, that is designed to capture practice changes [66].

The questionnaire will be structured to evaluate the Knowledge (multiple choice and binary questions [true or false and yes or no]), Attitudes (Likert scale), and Practices (simulated video scenarios with rubric), referred to as the Knowledge, Attitudes, and Practices (KAP) framework. The KAP framework allows for the exploration of a population to assess knowledge (what is known), attitudes (what is believed), and practices (what is done) [65,67]. Additionally, these questions may identify knowledge gaps and behavioral patterns, aligning with the program’s goal of assessing the impact and informing future improvements [67]. The knowledge-based questions will be formulated at the conclusion of the phase 1 qualitative data collection.

To evaluate a change in collaborative practices, the evaluation of the program will integrate the validated 16-question IPEC competency self-assessment tool into the questionnaire [22,68]. This instrument provides a validated means of assessing interprofessional competencies by measuring the participants’ self-efficacy, in relation to the IPEC core competency framework [68]. The IPEC competency self-assessment tool is reliable and validated and can therefore be used to assess the development of the participants’ collaborative practice skills [68].

Changes in interprofessional practice will also be objectively assessed using predefined scoring rubrics, with standardized criteria applied to minimize interrater variability. This process will involve participants watching a simulated video, aligned with the program’s learning outcomes. The participants will be asked to provide a written response that will be graded using a standardized rubric before and after the intervention.

#### Content Validity

The construction and validation of the knowledge, attitudes, and practice aspect of the questionnaire will be developed in alignment with the instrument development model [69]. The IPEC questions are validated and therefore do not require further validation.

Content validity will be achieved through review of the questionnaire by experts across varying health professionals from the targeted 5 discipline groups [70]. This is not a statistical test; yet, it aims to ensure the questionnaire explores the contextual issues [70]. This process will remain iterative, and the questionnaires will be adapted until each expert agrees on the final set of questions.

The scoring rubrics for the assessed scenarios in the pre- and postquestionnaire, will be developed in alignment with the WHO curriculum guidelines for training health care providers in responding to IPV [63]. Interrater consistency will be supported through assessor collaboration and the use of standardized scoring criteria. Interrater reliability will be measured through the kappa statistic to ensure that the data collected correctly measure the variable prior to the implementation of the project [71]. There will be 2 independent assessors who will grade all data requiring scoring through the rubrics. This process will be refined by piloting the rubric before implementation.

#### Test-Retest Reliability

The test-retest reliability method will be integrated into the implementation of the project to measure the reliability of the validated questionnaire [70,72]. A randomized subgroup of 20‐30 health professionals will be sought to volunteer and answer the questionnaire, and 2 weeks later, repeat the same questionnaire to determine the stability and replicability of the questions [73].

#### Sample Size

A minimum of 42 participants in total, from the 5 or more health professional groups will be recruited (midwives, social workers, medical staff, perinatal mental health nurse midwives, and Aboriginal Maternal and Infant Care workers). There will be several offerings of the simulation workshops conducted, due to the capacity of 10 participants in each session. To avoid bias, the program sessions will continue until all 5 health professional groups have been represented in the data collection.

#### Data Analysis

Descriptive and inferential statistics will be acquired through the pretest-posttest data collection questionnaires [74]. To determine the significance of perceived change post education, inferential statistics (paired *t* test and McNemar) will be used through the application of SPSS software (IBM SPSS Statistics; version 27).

Standard practice for reporting quantitative data includes detailing the baseline characteristics of participants; therefore, the protocol will report participant baseline characteristics descriptively (descriptive statistics) [75]. As attrition at the 4-month postintervention follow-up is anticipated, a table of baseline participant characteristics will be presented to allow comparison between participants retained in the study and those lost to follow-up [75]. This process thereby supports the assessment and analysis of potential attrition-related bias. To minimize missing data, questionnaires administered through REDCap (Research Electronic Data Capture; Vanderbilt University) will have the mandatory response settings enabled to reduce the risk of nonresponse bias [76]. Automated reminders regarding outstanding follow-up requirements will be sent to participants through the REDCap platform as a means of retention and tracking procedure recommendations [76].

The KAP survey will be structured to evaluate the knowledge, attitudes, and practices of participants. While the attitudes and practices are subjective self-reported changes, the knowledge-based questions will objectively measure the effect. Data that measure participant knowledge will be analyzed using the McNemar test, which is appropriate for paired categorical (dichotomous) data collected from the same participants pre- and postintervention. The McNemar test focuses on the discordant pairs to determine change for an individual participant over time [77]. Binary outcomes, with one categorical dependent variable (agree or disagree or true or false responses), will be compared to determine the effect, with *P* values calculated to assess the statistical significance [78]. The multiple-choice questions that are correct will receive a score of 1; incorrect and unsure will be scored 0 and will also be assessed at an item level using the McNemar test.

The IPEC tool and attitudes and practice Likert scale responses will be summed to generate a total score and domain-specific scores; higher scores indicate greater self-reported competency. The participant self-reported changes for each domain, and overall scores will be evaluated using a 2-tailed, paired *t* test to determine the mean and SD over time [78]. The Wilcoxon signed-rank test, however, will compare individual ordinal data for each Likert question to compare and determine the statistical significance.

The assessment of the video scenario questions will be scored in ordinal domains based on the rubrics and compared pre- and postinterventions using the Wilcoxon signed-rank test. The total rubric score will be analyzed through a paired *t* test.

Multiple comparisons arise from testing more than one outcome through different methods. Given the exploratory nature of the project, there will be no formal adjustment to multiple comparisons applied; instead, the results will be interpreted cautiously with consideration of the effect sizes and CIs. All data will be displayed in tables.

#### Triangulation of Findings

This project has both quantitative and qualitative data due to the nature of mixed methods research [23]. Each subset of data from the phases will be analyzed separately to draw meta-inferences in the analysis phase. Following the analysis, these meta-inferences will be triangulated in the interpretation stage of data integration. The triangulation process will adhere to the “triangulation protocol” outlined in O’Cathain et al [79]. The data will be displayed in a convergence coding matrix. Data will be presented in a table to demonstrate where an agreement has been identified and the level of convergence will be presented as either agreement, partial agreement, silence, or dissonance [79]. The triangulation protocol is of particular importance to determine which meta-inference the data are categorized in and therefore allowing for analysis of the strengths and weaknesses of the different methodologies [79].

## Results

This research incorporates both qualitative and quantitative approaches through an exploratory research design to guide the development of a simulation-based interprofessional workshop for health professionals. Phase 1 data are qualitative; however, these are drawn from separate cohorts of participants. Interviews with women with lived experience of IPV throughout the perinatal context will be undertaken. Concurrently, focus groups with health professionals who provide care to women in pregnancy will occur. These findings will be analyzed, and recommendations drawn, to integrate into the development of the ALIGN simulation workshop, which comprises phase 2 of the study. There will be no participant involvement in phase 2. Phase 3 is therefore where the quantitative data will occur through a single-group quasi-experimental pretest-posttest design. As the survey will be disseminated online through REDCap, an online web-based platform, the CHERRIES (Checklist for Reporting Results of Internet E-Surveys) checklist will be integrated into the reporting of the process. This will guide the chief investigator to report essential information about the survey design, development, recruitment, and data analysis processes, aiming to improve the project's quality and transparency [80]. The focus of phase 3 is to determine the perceived impact, through statistical analysis, of health professionals’ collaboration, knowledge, and behavioral responses to IPV disclosures in the perinatal context.

## Discussion

### Principal Findings

The paper outlines a proposed mixed methods research project that aims to design, implement, and evaluate an evidence-informed, simulation-based, interprofessional education intervention. Through the integration of input from women with lived experience of IPV in the perinatal context and the health care providers regarding available education and care provision, it is aimed that the workshop will improve health care professionals’ collaborative response to disclosures and ongoing management of care in the perinatal period. Phase 1 data are primarily qualitative and inform the workshop development. Phase 2 does not have participants or data collection methods. Quantitative data are collected in phase 3 with the aim of evaluating the perceived impact of the workshop. The integration of qualitative and quantitative data, therefore, seeks to provide a deeper understanding of the issues through the triangulation of data in a final publication.

### Strengths and Limitations

It is reasonable to suggest that the use of a mixed methods approach is a strength of the study. In addition to this, while the project is not co-designed, there has been careful consideration of how to ethically integrate the experiences, perspectives, and voices of women impacted by IPV. The project is supported by an advisory group to guide the researchers to approach the research in a trauma-informed manner. While the study has not commenced, it could be argued that the generalizability of the study may be limited through the initial evaluation process as the cohort is situated in South Australia.

### Comparison to Prior Work

IPV programs have been evaluated and published, yet there tends to be a focus on the observation of practice and knowledge changes, from a siloed profession [81,82]. This project leverages the insights from previous works and places an emphasis on exploring the perceived impact and changes in the health professional’s collaborative response.

There have been several studies undertaken in undergraduate education, with a siloed discipline approach [83-85] or alternatively, incorporating multiple health disciplines [18,19,21]. For example, a simulation workshop integrated into nursing and social work students’ curricula explored the impact of the simulation on students’ knowledge, perceptions of IPV, and their confidence in acting on disclosures during an interprofessional scenario [19]. The study demonstrated increased confidence in working with people with lived experience of IPV, attributing this finding to the interprofessional education [19]. The data, however, lacked measurable indicators to draw conclusions on the impact on collaboration between disciplines [19]. This highlights the need for an extension of interprofessional IPV programs to evaluate how interprofessional education influences collaboration between disciplines when caring for women with lived experience of IPV across the maternity sector.

### Dissemination Plan

The project is part of the chief investigator’s doctoral studies and therefore dissemination will occur through a thesis. Planned dissemination of the project also includes the publication of 5 manuscripts in academic journals. At the conclusion of the project, it is aimed that the workshop could potentially be implemented across broader settings throughout Australia.

### Conclusion

The development of interprofessional IPV education is integral to improving health care professionals’ behavior and responses. The outcomes of this project will provide valuable insights into the current context of health care for victim-survivors accessing health care in South Australia, in contrast to the perspective of health professionals. The findings then develop a simulation workshop. We anticipate that the insights from the evaluation of the simulation workshop will improve the collaborative response of health professionals, to therefore improve care received by victim-survivors.

## Supplementary material

10.2196/86289 Multimedia Appendix 1 An exemplar of the self-reflective questionnaire that all participants are asked to complete prior to engaging with the research.

10.2196/86289 Multimedia Appendix 2 Distress protocol: this is a guideline for management of participants if emotional distress arises throughout the interview, focus groups, or survey.

10.2196/86289 Multimedia Appendix 3 Pamphlet: a “thank you” for engagement pamphlet provided to all participants at the conclusion of the workshop. This pamphlet provides an overview of immediate and ongoing psychological support services.

## Abbreviations

ALIGN: Advancing Learning in Gender-Based Violence through Interprofessional Networks
CHERRIES: Checklist for Reporting Results of Internet E-Surveys
IPEC: Interprofessional Education Collaborative
IPV: intimate partner violence
KAP: Knowledge, Attitudes, and Practices
NHMRC: National Health and Medical Research Council
PIS: Participant Information Sheet
REDCap: Research Electronic Data Capture
WHO: World Health Organization
