# Supplementary material for: Strengthening Health Care Professionals’ Collaborative Responses to Women Experiencing Intimate Partner Violence in Pregnancy: Protocol for an Exploratory Mixed Methods Study
Source: JMIR Res Protoc. 2026 Mar 24;15:e86289. doi: 10.2196/86289 (PMC13012234; doi:10.2196/86289)
Supplement: Multimedia Appendix 1 [file resprot-v15-e86289-s001.docx]

**Self-Reflection Questions**

**Readiness to undertake the work**

- What are my reasons for wanting to become a participant on this project?
- Do I really want to become involved or am I feeling that I should?

**Resources needed**

- Do I have enough resources in place both personally and professionally to prevent my health and wellbeing being negatively impacted?
- What support might I need if my health and wellbeing is negatively impacted?
- What strategies will I use if someone (could be a research participant I am interviewing for example) reacts negatively or judgmentally?

**Safety considerations**

- Is it safe for me to become involved?
- Are there any ongoing risks posed by the person/s who abused me?
- Are there protections that can be put in place to increase my safety?

**Boundaries**

- How will I ensure my personal and professional boundaries are upheld?
- What are my personal limits regarding what I am happy to contribute?

**Legal considerations**

- Am I involved in any ongoing legal proceedings that may be jeopardised?
- Are there any potential legal consequences for me becoming a participant on this research?

**Other considerations**

- Are there people in my life who need to be aware of my decision to become a participant of this research?
- Am I clear about how I would make complaints, adjustments or provide feedback?
