# Supplementary material for: Strengthening Health Care Professionals’ Collaborative Responses to Women Experiencing Intimate Partner Violence in Pregnancy: Protocol for an Exploratory Mixed Methods Study
Source: JMIR Res Protoc. 2026 Mar 24;15:e86289. doi: 10.2196/86289 (PMC13012234; doi:10.2196/86289)
Supplement: Multimedia Appendix 2 [file resprot-v15-e86289-s002.pdf]

Participants will be asked to complete the self-reflective questions from Lamb et al (2023, p. 12).

- *"Do I have enough resources in place both personally and professionally to prevent my health and wellbeing being negatively impacted?"*
- *"How will I ensure my personal and professional boundaries are upheld?"*
- *"Do I really want to become involved or am I feeling that I should?"*

FOCUS GROUP | **INTERVIEWS** | **SBLE WORKSHOP**

ASSESS

The participant indicates signed of distress when engaging.

"I can see that (link into what signs of distress the participant may be demonstrating). Would you like me to pause? Or would you like to take a moment and stop?"

YES

PAUSE AND REASSESS

STOP

Offer immediate support and assess the mental status of participant:

How are you feeling right now?
Are you feeling safe?
I can wait until you feel comfortable to recommence. Just let me know when that may be"

On the questionnaire, embedded into RedCAPP, there will be strategically placed statements throughout to 'check in'. Questions such as "*are you okay to proceed with the questionnaire?*" will be integrated at several checkpoints for the participant. This is a non-verbal way of checking in with participants when you are not physically present.

NO

CHECK IN

Now that the interview is finished, I just wanted to check in and see if there was any support I could offer?

Further Questions:
If you are unsure whether the participant feels comfortable in continuing, ask:

Would you prefer to stop the interview?

NO

YES

STOP

Discontinue

Can I help connect you with someone to help support you now?

Ask the participant if they would like me to contact any family members or support people

YES

RESOURCES

1800RESPECT
Lifeline 13 11 14
Beyond Blue: 1300 224 636
13 Yarn: 13 92 76

Ensure the research student (Carly Jones) and the Primary Supervisor (Angela Brown) have been contacted.

If the participant withdraws from the SBLE workshop, follow up in the questionnaires will not occur. The participant will be ineligible to undertake phase three.
