# Supplementary material for: Strengthening Health Care Professionals’ Collaborative Responses to Women Experiencing Intimate Partner Violence in Pregnancy: Protocol for an Exploratory Mixed Methods Study
Source: JMIR Res Protoc. 2026 Mar 24;15:e86289. doi: 10.2196/86289 (PMC13012234; doi:10.2196/86289)
Supplement: Multimedia Appendix 3 [file resprot-v15-e86289-s003.pdf]

Making a Difference Together

Sometimes taking part in research can bring up unexpected emotions or memories, whether connected to your own experiences or those of others. This is a normal response. If you notice difficult feelings, it may help to take some time for yourself, talk with someone you trust, or reach out for support if you need it.

Please remember that you can contact the PhD student or Primary supervisor.

Carly Jones

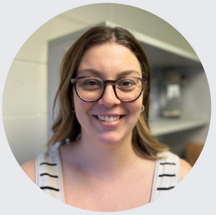

PhD student
8302 2160
carly.jones@unisa.edu.au

Angela Brown

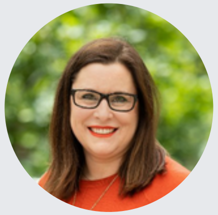

Primary Supervisor
8302 2897
angela.brown@unisa.edu.au

ETHICS NUMBER ###
Protocol number 207340
University of South Australia *(or Adelaide University by the time* of dissemination)

If needed, ongoing support can be accessed through specialist services listed below:

Yarrow Place: 1800 817 421

Pregnancy Advisory Centre (PAC) Counselling services: (08) 7117 8999

Victim support services: 1800 842 846

Women's Safety Services SA: (08) 8152 9200

Migrant Women's Support Program: (08) 8152 9260

Family & Domestic Violence (Relationships Australia) 1300 364 277

Domestic Violence and Aboriginal Family Violence Gateway Service 1800 800 098

Every voice adds to a clearer understanding of how people and communities are affected by relationships, safety, and wellbeing. By taking part, you are helping researchers and practitioners learn more, so that future support, services, and policies can be stronger and more responsive. Your involvement is part of creating positive change.

We are deeply grateful for the time and thought you have given to this research. By sharing your experiences and perspectives, you are helping to build knowledge that can make a difference for individuals, families, and communities. Your contribution is valued and will play a part in shaping safer, healthier futures.

Where to seek immediate support?

Beyond Blue: 1300 22 4636

Lifeline: 13 11 14

Mental Health Triage: 13 14 65

13 YARN: 13 92 76

1800 Respect: 1800 737 732

Domestic Violence Crisis Line: 1800 800 098

Full Stop Australia: 1800 385 578

National Domestic & Sexual Violence 1800 737 732
